# Antibacterial mechanism of rhodomyrtone involves the disruption of nucleoid segregation checkpoint in *Streptococcus suis*

**DOI:** 10.1186/s13568-020-01047-x

**Published:** 2020-06-08

**Authors:** Apichaya Traithan, Pongsri Tongtawe, Jeeraphong Thanongsaksrikul, Supayang Voravuthikunchai, Potjanee Srimanote

**Affiliations:** 1 Graduate Program in Biomedical Sciences, Faculty of Allied Health Sciences, Thammasat University, Khlong Nueng, Khlong Luamg, Pathumthani 12120 Thailand; 2 Excellent Research Laboratory on Natural Products, Department of Microbiology, Faculty of Science and Natural Product Research Center of Excellence Prince of Songkla University, Songkhla, 90110 Thailand

**Keywords:** *Streptococcus suis*, Cell division, Rhodomyrtone, Cell division defects

## Abstract

Rhodomyrtone has been recently demonstrated to possess a novel antibiotic mechanism of action against Gram-positive bacteria which involved the multiple targets, resulting in the interference of several bacterial biological processes including the cell division. The present study aims to closely look at the downstream effect of rhodomyrtone treatment on nucleoid segregation in *Streptococcus suis*, an important zoonotic pathogen. The minimum inhibition concentration (MIC) and the minimum bactericidal concentration (MBC) values of rhodomyrtone against the recombinant *S. suis* ParB-GFP, a nucleoid segregation reporter strain, were 0.5 and 1 µg/ml, respectively, which were equivalent to the potency of vancomycin. Using the fluorescence live-cell imaging, we demonstrated that rhodomyrtone at 2× MIC caused incomplete nucleoid segregation and septum misplacement, leading to the generation of anucleated cells. FtsZ immune-staining of rhodomyrtone-treated *S. suis* for 30 min revealed that the large amount of FtsZ was trapped in the region of high fluidity membrane and appeared to be able to polymerize to form a complete Z-ring. However, the Z-ring was shifted away from the midcell. Transmission electron microscopy further confirmed the disruption of nucleoid segregation and septum misplacement at 120 min following the rhodomyrtone treatment. Asymmetric septum formation resulted in either generation of minicells without nucleoid, septum formed over incomplete segregated nucleoid (guillotine effect), or formation of multi-constriction of Z-ring within a single cell. This finding spotlights on antibacterial mechanism of rhodomyrtone involves the early stage in bacterial cell division process.

## Introduction

*Streptococcus suis* is the Gram-positive bacterium causing the severe systemic infections in young and weaning piglets (Halaby et al. 2000; Lun et al. 2007). Zoonotic transmission of this pathogen to human occurs via direct contact with the sick pigs or consumption of contaminated meat and pork products (Segura et al. 2014; Wertheim et al. 2009). Similar to *Streptococcus pneumoniae* infections, penicillin and ampicillin previously were the mainstay of treatment of *S. suis* infections (Lakkitjaroen et al. 2011; Yu et al. 2018; Zhang et al. 2015). To date, the efficacy of these antibiotics was seriously compromised as evidenced by the frequent isolation of ampicillin-resistant *S. suis* strains from infected swine and human (Yu et al. 2018; Zhang et al. 2015). Therefore, novel and effective antimicrobial agents are indeed needed for the management of *S. suis* infection.

Rhodomyrtone is a principle antimicrobial compound found in ethanoic extract of medicinal plant *Rhodomyrtus tomentosa* (Aiton) *Hassk*. Leaves (Leejae et al. 2013; Limsuwan et al. 2009). Its antibacterial potency towards the Gram-positive bacteria is equivalent to that of top available drugs. A molecular antibacterial mechanism underlining rhodomyrtone activity has been mostly elucidated. Integrative proteomic and metabolomic analyses revealed the effects of rhodomyrtone on pneumococcus carbohydrate metabolism. It caused the reduction of capsule biosynthesis and formation (Mitsuwan et al. 2017). In addition, transcriptomic analysis of rhodomyrtone-treated methicillin-resistant *Staphylococcus aureus* (MRSA) showed that the compound had both immediate and late effects on MRSA gene expression. Bacterial cell cycle maintenance (*scd*) and bacterial septum formation (*ftsZ*) were among the 35 down-regulated genes (Sianglum et al. 2012). Furthermore, the effects of rhodomyrtone on MRSA cell wall alterations, abnormal septum formation, and aberrant cell morphology have been visualized by transmission electron microscopy (Sianglum et al. 2011, 2012). Recently, it has been demonstrated that rhodomyrtone treatment as early as 30 min could increase MRSA membrane permeabilization, alteration of cell wall, and cell membrane integrity. As a consequence, cytoplasmic component leakage, cell lysis, and bacterial cells with undefined septum were observed (Sianglum et al. 2018). In silico analysis using molecular docking revealed that rhodomyrtone was able to bind to FtsZ and changed its conformation. This was further confirmed by the partial loss of GTPase activities and FtsZ polymerization in vitro (Saeloh et al. 2017). More recently, rhodomyrtone was clearly demonstrated to be a membrane-active compound. Following the treatment, *S. aureus* membrane potential decrease immediately at low doses resulted in releasing of ATP and cytoplasmic protein without pore-formation effect (Saising et al. 2018). Other workers demonstrated the increase in membrane fluidity and the collapsed membrane potential in *Bacillus subtilis* as well as the relocalization of seven membrane proteins (FtsA, DivIVA, MinD, PlsX, MreB, MurG and SdhA), trapping within the region of increased lipid fluidity (RIF) (Saeloh et al. 2018). Accumulation of high concentration of FtsA and other divisome proteins suggested that they might interfere with the dynamics of cell division complex that need to position at the midcell area in timely and orderly manner. However, it has not yet known how these structural and physical changes as well as membrane potential collapsed directly affected the mechanisms of cell division or changes in cell morphology.

Chromosome or nucleoid segregation is an efficient process which ensures that the bacterial daughter cells inherit the genetic materials (Hajduk et al. 2016; Lewis 2001). It was proposed that the nucleoid segregation mechanism could be driven by the forces of bacterial general processes such as DNA replication and transcription and/or DNA-interacting proteins (Dworkin and Losick 2002; Toro and Shapiro 2010). ParABS system has been documented to play an important role in bacterial nucleoid segregation (Kjos and Veening 2014; Lewis 2001; Toro and Shapiro 2010; Lemon  and Grossman 2001). It consists of ParA, a walker type ATPase, and ParB, partitioning protein that bind to the specific DNA sequences, *parS*-sites, located at proximal to *ori* region. *B. subtilis* possesses a completed partitioning system (Ireton et al. 1994; Wang et al. 2014) while *Streptococcus pneumoniae* carries only the ParB protein and the *parS*-sites (Attaiech et al. 2015; Kjos and Veening 2014). The absence of ParB in both organisms resulted in the significant nucleoid segregation defect, leading to 1–4% anucleated cells (Kjos and Veening 2014; Lee et al. 2003; Minnen et al. 2011). In addition, the disruption of pneumococcus transcription by rifampicin or streptolydigin treatment resulted in the nucleoid segregation defects (2–3% anucleated cells) (Kjos and Veening 2014). Other antibiotics such as quinolone and A22, inhibitors of the bacterial cell wall protein, MreB, have been demonstrated to inhibit the nucleoid segregation in *Escherichia coli* (Kruse et al. 2003). This study spotlights on the antibacterial mechanism of rhodomyrtone involves the disruption of bacterial nucleoid segregation checkpoint leading to the cell division defects.

## Materials and methods

### Bacterial strains, plasmids, and growth conditions

Serotype 2 *S. suis* reference strain P1/7 was isolated from blood of dying pig with meningitis (Clifton-Hadley 1984). *S. suis* was grown on Columbia blood agar plate (BA) supplemented with 5% red cells at 37 °C under 5% CO_2_ for 24 h. A single colony was inoculated into Todd-Hewitt broth (THB) and incubated at 37 °C, 5% CO_2_ overnight. *E. coli* DH5α was used as a host for cloning and plasmid propagation. The strains were grown in Lubria-Bertani (LB) broth and agar at 37 °C and supplemented with spectinomycin (100 µg/ml) when required.

### Construction of recombinant *S. suis* ParB-GFP

In order to visualize nucleoid segregation, *S. suis* strain P1/7 carrying *gfp* fusion to 3′-end of *parB* gene was constructed such that ParB expression was driven using its endogenous promoter. The four overlap extension primers (Additional file 1: Table S1) were designed to fuse *gfp* coding sequence to the 3′-end of *parB*. The *parB* and *gfp* amplicon was purified to use as a megaprimer to amplified *parB::gfp* fusion amplicons. After several round of thermocycles, the outer primers, Us*parB*-F/Ds*parB*-R were added to the reaction and thermocycles were continued to 35 cycles. The plasmid DNA of pSET4s, thermosensitive suicide vector, and overlap extension PCR product, *parB::gfp*, were purified and endonuclease digested with *Sph*I and *Bam*HI. The digested vector and amplicon were ligated and transformed into the competent *E. coli* DH5α host. *E. coli* transformant carrying pSET4s*::parB::gfp* was selected on LB agar supplemented with 100 µg/ml spectinomycin. The *E. coli* clones carrying pSET4s*::parB::gfp* were screened by colony PCR using Us*parB*-F*/*Ds*parB*-R and pSET4s-F/R primer pairs (Additional file 1: Table S1). The correct *parB::gfp* fusion was verified by DNA sequencing analysis. Competent *S.*  *suis* cells were prepared by the addition of competence-inducing peptide (ComS), as previously described (Zaccaria et al. 2014). The purified pSET4s*::parB::gfp* plasmid was then transformed into *S.* *suis*. The recombinant *S.* *suis* carrying pSET4s*::parB::gfp* (recombinant *S.* *suis* ParB-GFP) was selected on THB supplemented with 50 µg/ml spectinomycin. The presence of *parB::gfp* was detected by PCR and expression of ParB-GFP in the recombinant *S.* *suis* was visualized by epifluorescence microscopy (Olympus BX53 microscope).

### Determination of rhodomyrtone minimum inhibitory concentration (MIC) and minimum bactericidal concentration (MBC) against *S.* *suis*

Overnight cultures of wildtype *S. suis* and recombinant *S.* *suis* ParB-GFP were used to adjust the turbidity ca. 1.5 × 10^8^ CFU/ml using McFarland standard no. 0.5. The bacterial suspension was further adjusted in fresh Muller Hilton broth (MHB) to reach ca. 10^6^ CFU/ml. The MIC and MBC values of purified rhodomyrtone against *S.* *suis* were determined using broth microdilution method according to CLSI guidelines (CLSI 2018). In brief, rhodomyrtone was dissolved in 100% DMSO and two-fold serially diluted in MHB in wells of 96-wells microtiter plate to generate a final concentration ranging from 128 to 0.0625 µg/ml. An equal volume (100 µl) of the bacterial suspension (ca. 10^5^ CFU) was added into each well and further incubated at 37 °C under 5% CO_2_ for 18 h. The MIC determination of rhodomyrtone against *S.* *suis* P1/7 and ParB-GFP was carried out in triplicate wells. The MIC was interpreted following the three separate experiments. Thereafter, MBC_99_ of bacteria was further determined by plating out on BA to enumerate the colonies.

### Time-kill assay

The ca. 10^5^ CFU *S.* *suis* P1/7 and ParB-GFP were treated with MHB supplemented with rhodomyrtone at 2× (1 µg/ml), 1× (0.5 µg/ml), 0.5× (0.25 µg/ml), 0.25× (0.125 µg/ml), and 0.125× (0.0625 µg/ml) and incubated at 37 °C. Samples were collected every hour for 8 h and once at 24 h. Surviving bacteria were enumerated on BA. A tube containing 1% DMSO was used as a growth control. The experiment was performed in triplicates.

### Determination of GFP activity of recombinant *S.* *suis* ParB-GFP

Recombinant *S.* *suis* ParB-GFP was grown in THB at 37 °C until an OD_600 nm_ reach to ~ 0.2. Cells were subsequently treated at 0.0625 to 1 µg/ml rhodomyrtone or 1% DMSO (negative control). Unless otherwise stated, samples were collected at 15 min intervals and ParB-GFP signals were measured by using spectroUVmeter (Thermo Electron Corporation, USA).

### Investigation of *S. suis* nucleoid segregation dynamic by fluorescence microscopy

Recombinant *S.* *suis* ParB-GFP was grown in THB without antibiotics until an OD_600 nm_ ~ 0.2. Cells were continuously grown and collected every 15 min to investigate the *S. suis* nucleoid segregation dynamic before treatment. Treated cells were subsequently treated at 0.0625 to 1 µg/ml (0.125 to 2× MIC) rhodomyrtone and collected every 15 min intervals. Quinolone was used as DNA replication inhibitor (Georgopapadakou and Bertasso 1991). Rifampicin (RNA synthesis inhibition antibiotic) was used as nucleoid segregation inhibitors as previously described (Dworkin and Losick 2002; Kjos and Veening 2014). 1% DMSO was a negative control. Cells were immobilized on a thin film of 1.2% agarose and immediately observed using an Olympus BX53 microscope equipped with a Photometrics CoolSNAP fx digital camera. Image acquisition was performed using ImageJ software and processed with Adobe Photoshop 6.0.

### Immunofluorescence microscopy

Immunostaining was adapted from Morlot et al. (2003). Briefly, exponential recombinant *S.* *suis* ParB-GFP grown in the presence of 1 µg/ml rhodomyrtone (2× MIC) or rifampicin or 4 µg/ml quinolone for 30 min. Cells were harvested by centrifugation at 10,000×*g* for 5 min, fixed in 4% paraformaldehyde for 15 min followed by 45 min incubation on ice. Cells were washed three times in PBS and resuspended in GTE buffer (50 mM glucose, 20 mM Tris–HCl pH 7.5, 10 mM EDTA) and treated with lysozyme (final concentration 0.1 mg/ml). Cells were immediately transferred onto poly l-lysine coated cover slips, washed with PBS and air-dried. It was then soaked in methanol at − 20 °C for 5 min, air dried and rehydrated with PBS. The cover glasses were blocked for 30 min with 2% (w/v) bovine serum albumin in PBS (BSA-PBS), incubated for 1 h with 1:200 dilutions of rabbit anti-FtsZ antibodies (kindly provided by Professor Cécile Morlot, Institut de Biologie Structurale (IBS), Grenoble, France), washed five times with PBS, and further incubated with Alexa fluor 594 conjugated goat anti-rabbit immunoglobulins G (Fermentus, USA.), in BSA-PBS. For visualization of bacterial chromosomal DNA, 1 µg/ml of Hoechst 33342 dye (Sigma, USA.) was included with the secondary antibody. Following extensive washing with PBS, cover glasses were observed and photographed under a Leica DM IRB fluorescence microscope equipped with a 63×\Bertrand immersion objective and standard filter sets for visualizing DAPI, FITC and Alexa fluor 594. Images were captured with a DC350 F digital camera system (Leica) driven by the Qfluoro software package (Leica). Rabbit anti-FtsZ antibodies was omitted in the secondary antibodies control.

### Transmission electron microscopy

Exponential recombinant *S.* *suis* ParB-GFP grown in the presence or the absence of rhodomyrtone at 1 µg/ml (2× MIC) or 0.25 µg/ml (0.5× MIC) for 30 min and 120 min, respectively. Cells were harvested by centrifugation at 10,000×*g* for 5 min. The cells were washed twice, resuspended in PBS, and fixed with 2.5% glutaraldehyde in sucrose phosphate buffer (PB) overnight. Cells were then recovered by centrifugation, washed twice with PB and then fixed with 1% osmium tetraoxide at room temperature for 1 h. The samples were dehydrated with gradient ethanol solutions (30% to 90% at 10 min intervals) and incubated twice in 100% ethanol for 15 min. Bacterial cells were then embedded in Epon 812 resin, polymerized for 2 days, cut into 90 nm-thin slices, placed on grid and counter stained with uranyl acetate and lead citrate. Cellular contents and morphology were observed at 200 nm magnification and photographed by a Hitashi H7000 transmission electron microscope at the Department of Tropical Pathology, Faculty of Tropical Medicine, Mahidol University.

## Results

### Identification of gene involving in *Streptococcus suis* nucleoid segregation

**Fig. 1:**
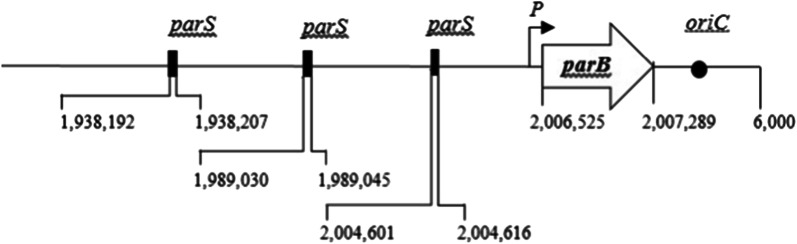
Schematic diagram represents genetic organization of *Streptococcus suis* nucleoid segregation machinery. Locations of *parB* and *smc* genes (white block arrows), *parS* (black rectangles), putative promoter (*P*), and *oriC* (black circles) were shown. The digits below the figure indicated the number of nucleotide sequence in base pairs (bp)

In this study, DNA region involving in nucleoid segregation system of *S.* *suis* was identified from genome sequence of P1/7 reference strain (Genbank Accession number NC_012925). By using GenSkew application (http://mips.gsf.de/services/analysis/genskew), the putative 202 AT-rich nucleotides segment of chromosomal origin of replication (*oriC*) region containing 4 DnaA boxes was predicted to locate at the position of nucleotide numbers (nt) 2,007,290 to 2,007,491. BLASTN and BLASTP revealed that *S.* *suis* nucleoid segregation system genes contained only *parB* located in the vicinity of *oriC* (nt 2,006,525 to 2,007,289) characteristic genome topology of *parB. parS*. *S.* *suis parB* was previously annotated as SSU_RS09915 in genome of P1/7 *S. suis*. Approximately 3 kb region containing *parB* was amplified from genomic DNA of *S.* *suis* P 1/7 and subjected to nucleotide sequencing. BlastX analysis (Additional file 1: Fig. S1) revealed that *S.* *suis* ParB were 765 nucleotides long. Its deduced amino acid sequence (254 residues) had 54% identity to the well-characterized ParB from D39 *S.* *pneumoniae* (Genbank Accession number NC_008533). *S.* *suis* ParB completely carried two conserved ParB motifs, DNA binding (amino acid residues 4 to 100), and activating ATPase activity (residues 7 to 95). Three stretches of *S.* *suis parS* consensus sequences, NGTTTCACGNNAAACN, were identified in an anticlockwise regions of *S.* *suis oriC* at − 2.8 kbp (− 0.5°), − 18 kbp (− 3.24°), and – 9 kbp (− 12.42°) (Fig. 1). The three sequences exhibited 92.19% identity to entire *parS* from *Streptococcus* spp. including *S.* *pneumoniae* (Additional file 1: Fig. S2). The data obtained in our study further emphasized that genetic components of nucleoid segregation system were highly conserved among *Streptococcus* spp. including *S.* *suis*.

### Dynamic of nucleoid segregation in *Streptococcus suis*

**Fig. 2:**
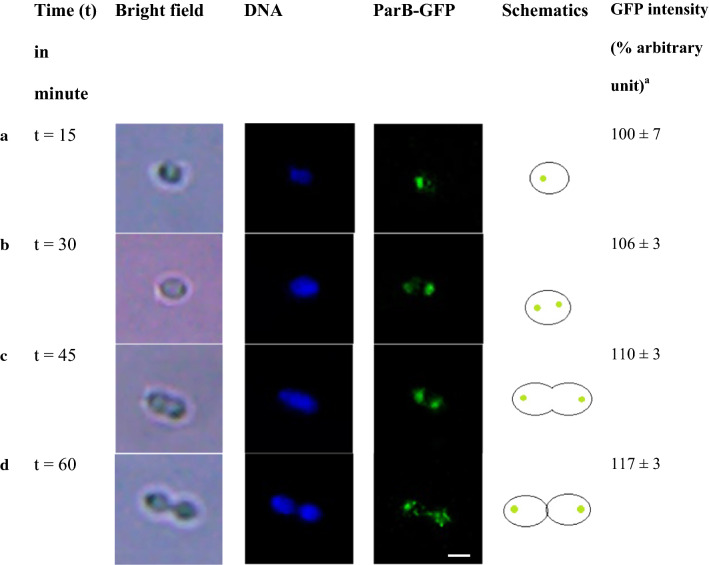
Localization of *Streptococcus suis oriC* (ParB-GFP) in representative cells at the different stages of the division was visualized by fluorescence microscopy. **a**–**d** Designated arbitrary time points of 15 min intervals (t = 0 to 60 min). Recombinant *S. suis* ParB-GFP cells were spontaneously expressed ParB-GFP (green) and were stained to visualize DNA (blue). Scale bar = 2 µm. ^a^The quantitation of ParB-GFP activity as determined by fluorescence signal was reported in arbitrary units

To investigate *S.* *suis* nucleoid segregation, ParB-GFP (Green fluorescent protein) was used as a reporter to mark *parS* in order to indicate the location of the neighborhood *oriC* sequence. The dynamic of *S.* *suis* nucleoid localization during exponential phase was shown in Fig. 2a–d. ParB-GFP signal of the reporter strain exhibited as single foci representing the initiation of nucleoid replication at 15 min (t = 15, Fig. 2a). At 30 min, *S.* *suis* nucleoid was duplicated as two bright green ParB-GFP foci (or two *oriC*) in each cell pole of a single cell (t = 30, Fig. 2b). The nucleoid segregation was completed and Z-ring started to constrict within 45 min (t = 45, Fig. 2c). The septum was formed within 1 h as each *oriC* was localized to the newly generated daughter cells (t = 60, Fig. 2d). The data demonstrated that *S.* *suis* nucleoid segregation (i.e. timing from initiation of duplicated *oriC* to *oriC* mobilization to the newly generated daughter cells) was approximately 30 min, and *S.* *suis* cell division cycle (i.e. timing from initiation of duplicated *oriC* to beginning of the next round of *oriC* duplication) was 60 min. These observations were further confirmed by the gradual increase in GFP activity quantification during the division cycle (Fig. 2, right column). Our finding demonstrated that *S.* *suis* nucleoid was rapidly replicated and completely segregated to each cell pole using three quarters of the duration of the cell division cycle.

### Antibacterial activity of rhodomyrtone against *Streptococcus* *suis*

**Fig. 3:**
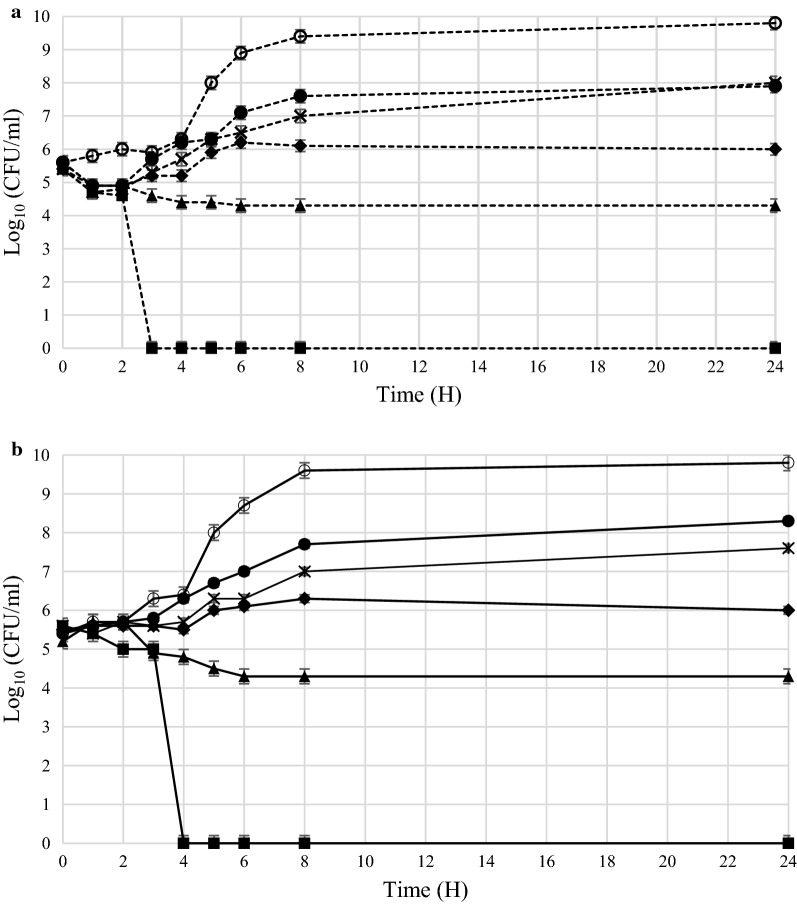
Time-kill curves of wildtype (**a**) and recombinant *Streptococcus* *suis* ParB-GFP (**b**) after treatment with rhodomyrtone at 1 µg/ml (filled square), 0.5 µg/ml (filled triangle), 0.25 µg/ml (filled diamond), 0.125 µg/ml (×), and 0.0625 µg/ml (filled circle). 1% DMSO (open circle) was used as a negative control. The results were shown as mean ± SD of three independent cultures

The MIC and MBC of rhodomyrtone, rifampicin, and quinolone against the wildtype parent P1/7 *S.* *suis* and the isogenic ParB-GFP reporter strains were shown in Table 1. MIC and MBC values of rhodomyrtone ranged from 0.5 and 1 µg/ml, which were equivalent to those of rifampicin, while MIC and MBC of quinolone were 2 and 4 µg/ml, respectively. The antibacterial efficacy of rhodomyrtone was further determined by time-kill assay. The bactericidal activity of rhodomyrtone was concentration and time dependence. In ParB-GFP *S.* *suis*, three log-fold decrease in cell numbers was evident within 4 h (Fig. 3b), compared with 3 h in the wildtype parent strain (Fig. 3a), following the treatment with 1 µg/ml rhodomyrtone.

**Table 1:** Minimal inhibitory concentration (MIC) and minimal bactericidal concentration (MBC) values of rhodomyrtone, rifampicin and quinolone against *Streptococcus suis* wildtype strain P1/7 and recombinant reporter strain ParB-GFP

Antibacterial agents	Antibacterial activity MIC/MBC (µg/ml)	
	*S. suis* wildtype strain P1/7	*S.* *suis* recombinant strain ParB-GFP
Rhodomyrtone	0.5/1	0.5/1
Rifampicin	0.5/1	0.5/1
Quinolone	2/4	2/4

### Effects of rhodomyrtone on *Streptococcus suis* nucleoid segregation

**Fig. 4:**
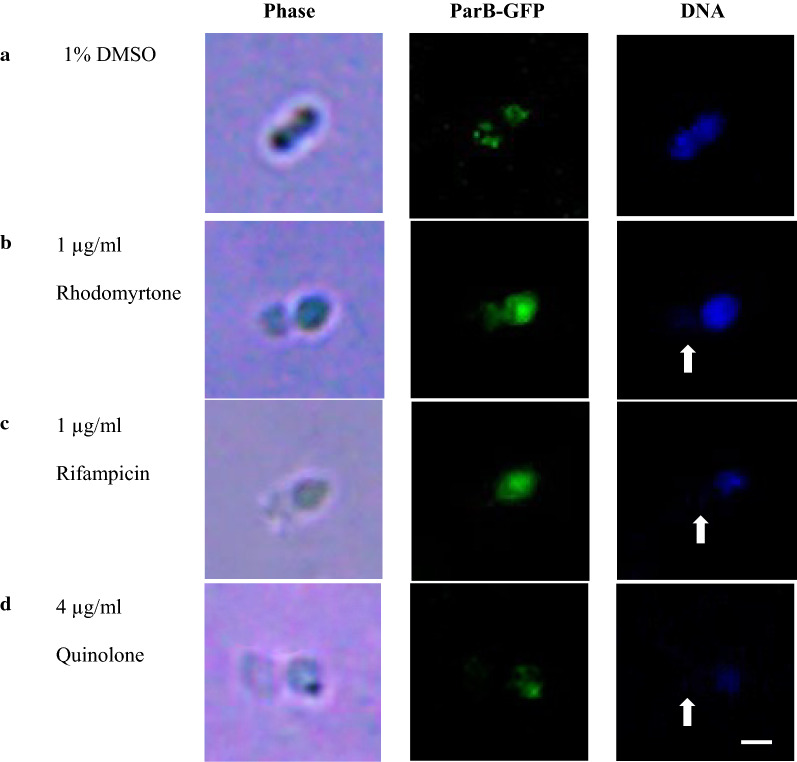
*oriC* localization (ParB-GFP) and the presence of anucleated cells following rhodomyrtone treatment was visualized by fluorescence microscopy. **a** 1% DMSO, **b** 1 µg/ml rhodomyrtone, **c** 1 µg/ml rifampicin and **d** 4 µg/ml quinolone for 30 min. Phase-contrast images (Phase), ParB-GFP (*oriC*) localization (green) and DNA (blue). Anucleated cells were indicated by white arrows. Scale bar = 2 µm

In order to investigate whether rhodomyrtone affected *S.* *suis* nucleoid segregation, recombinant *S.* *suis* ParB-GFP was treated with 0.0625 to 1 µg/ml rhodomyrtone. Fluorescent micrographs illustrated a successful nucleoid segregation with a mobilization toward cell poles in the negative control, 1% DMSO treatment (Fig. 4a). Treatment with either 1 µg/ml rhodomyrtone (Fig. 4b) or rifampicin (Fig. 4c) for 30 min resulted in the partial segregation of nucleoid as they failed to mobilize to the new cell pole indicating by non-segregated *oriC* (unnoticeable of separating ParB-GFP foci). In the treatment with 4 µg/ml of quinolone, only single ParB-GFP focus was observed as a consequence of DNA replication inhibition by quinolone (Fig. 4d). In addition, the quantitative measurement of ParB-GFP signals for rhodomyrtone, rifampicin, and quinolone was significantly decreased since the initial exposure (Additional file 1: Table S2, t = 0) suggesting that rhodomyrtone is likely to produce a stronger impact on ParB-foci dynamics than nucleoid duplication. The aberrant of the nucleoid segregation led to the appearance of 0.3 ± 0.2% of anucleated cells illustrated by the absence of DNA staining by Hoechst 33342 at 30 min post 1 µg/ml rhodomyrtone treatment (Fig. 4b and Table 2, t = 30). At this time point, no anucleated cells were found in the treatment with sub-lethal concentrations of rhodomyrtone (0.0625 to 0.5 µg/ml). Moreover, the two foci of ParB-GFP or *oriC*s were appeared to successfully mobilize to each of the cell poles indifference to that of the 1% DMSO-treated cells. Furthermore, we found that rhodomyrtone treatment could generate *S.* *suis* anucleated cells in the dose and time dependent manner as showed in Table 2. 1 µg/ml rhodomyrtone treatment yielded significantly higher numbers of anucleated cell (2.3 ± 0.2%, 3.4 ± 0.4% and 3.5 ± 0.4% at 60, 120 and 240 min post-treatment, respectively) (P-values < 0.05). The similar phenomenon was also observed in the treatment with sub-lethal concentrations of rhodomyrtone. Furthermore, 0.125 µg/ml rhodomyrtone was the lowest concentration to produce a significantly number of anucleated cells (Table 2, t = 240, P-values < 0.05). Anucleated cells found in positive control agents, rifampicin and quinolone treatment were also gradually increased in the time and concentration dependent manner (Table 2).

**Table 2:** Percentage of the average anucleated cell number in rhodomyrtone treated-recombinant *Streptococcus suis* ParB-GFP

Time (t) in minute	Percentage of average anucleated cell number (%)							
	1% DMSO	Rhodomyrtone (µg/ml)					1 µg/ml Rifampicin	4 µg/ml Quinolone
		1	0.5	0.25	0.125	0.062		
0	0	0	0	0	0	0	0	0
15	0	0	0	0	0	0	0	0
30	0	0.3 ± 0.2	0	0	0	0	0.7 ± 0.2*	0.9 ± 0.2*
45	0	0.3 ± 0.2*	0	0	0	0	1.1 ± 0.2*	2.1 ± 0.2*
60	0	2.3 ± 0.2*	1.4 ± 0.4*	0.7 ± 0.2*	0.1 ± 0.2	0	4.1 ± 0.6*	6.3 ± 0.2**
120	0	3.4 ± 0.4*	1.9 ± 0.2*	0.7 ± 0.2*	0.1 ± 0.2	0.1 ± 0.2	4.8 ± 0.4*	6.4 ± 0.2**
240	0	3.5 ± 0.4*	2.1 ± 0.2*	1.3 ± 0.2*	0.7 ± 0.2*	0.3 ± 0.2	5.2 ± 0.4*	6.6 ± 0.4*

Cells were exposed to 0.062 to 1 µg/ml rhodomyrtone, 1 µg/ml rifampicin, and 4 µg/ml quinolone. DNA was stained by Hoechst 33342 and visualized. Anucleated cells were determined by absence of Hoechst signal (blue). For each treatment, over 500 cells were counted. The results were shown as mean ± SD of three independent experiments

*The significant changes in anucleated cell numbers between different treatments and durations were compared to 1% DMSO negative control using the Student’s t-test. Significant differences were indicated as * (P-values < 0.05) and ** (P-values < 0.001)

### Effects of rhodomyrtone on *Streptococcus suis* septum formation

**Fig. 5:**
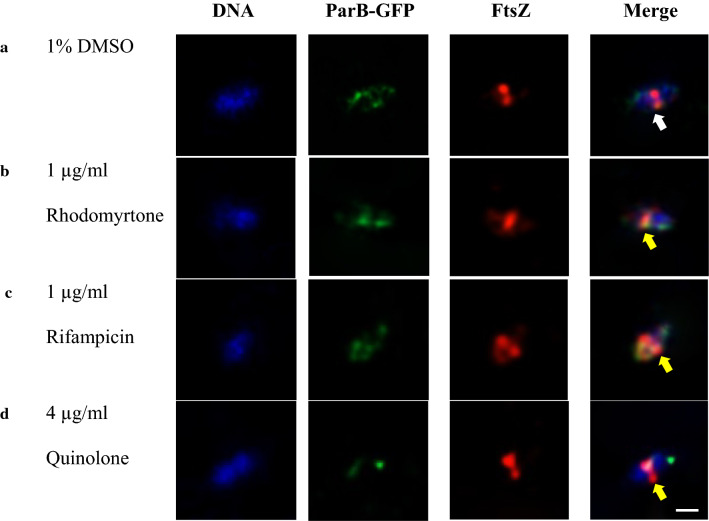
Effects of rhodomyrtone on recombinant *Streptococcus suis* ParB-GFP nucleoid and septum positioning were visualized by immunofluorescence microscopy. Representative *S.* *suis* after 30 min exposure to **a** 1% DMSO, **b** 1 µg/ml rhodomyrtone, **c** 1 µg/ml rifampicin, and **d** 4 µg/ml quinolone were shown. Localization of Hoechst 33342 stained DNA (blue), ParB-GFP (green), FtsZ (red) and overlaid of three fluorescence colors (Merge) were shown. Arrow indicated the characteristic of Z-ring (FtsZ) placement at midcell (**a**). Arrows indicated misplaced Z-ring shifting away from midcell found in rhodomyrtone, rifampicin, and quinolone-treated cells (**b**–**d**, respectively). Scale bars = 1 µm

To further investigate the effect of rhodomyrtone on *S. suis* septum formation, the septum position in rhodomyrtone-treated ParB-GFP *S. suis* was probed with anti-FtsZ antibody and visualized by fluorescence microscope. Immunofluorescence micrograph illustrated that in 1% DMSO control (Fig. 5a, arrow), *S.* *suis* completely achieved nucleoid segregation and septum was definitely placed at midcell of the so-divided cells. In contrast, treatment with 1 µg/ml rhodomyrtone for 30 min (Fig. 5b, arrow), *S.* *suis* nucleoid exhibited partially segregated and defect septum formation. Although, FtsZ was polymerized and completely formed constricted Z-ring, its position was misplaced by shifting away from midcell. Aberration of Z-ring placement phenotypes such as the accumulated FtsZ protein at one cell pole or dispersed and shifted away from the center were also found in rifampicin and quinolone treatment (Fig. 5c, d, arrows).

### Transmission electron microscopy

**Fig. 6:**
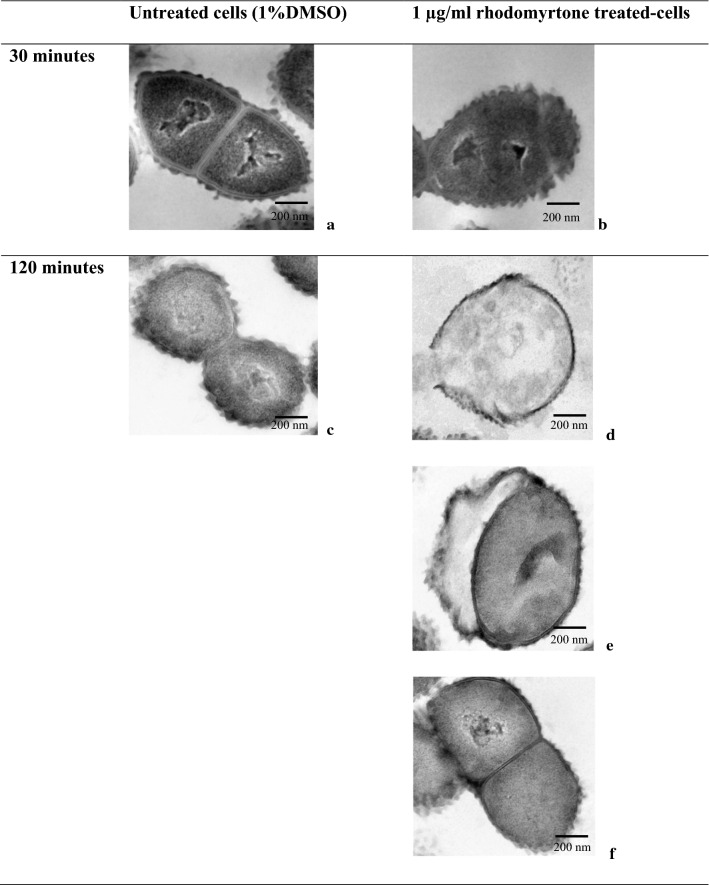
Transmission electron microscopy demonstrating the effects of rhodomyrtone on *Streptococcus suis* nucleoid segregation, septum formation and cell morphology. Recombinant *S. suis* ParB-GFP were treated with addition of 1 µg/ml of rhodomyrtone for 30 min (**b**) and 120 min (**d**–**f**). Cultures supplemented with 1%DMSO for 30 min (**a**) and 120 min (**c**) were used as untreated control culture. Black arrow indicated incomplete-segregated nucleoid. Scale bar = 200 nm

*Streptococcus suis* cytoplasmic content and morphology following the rhodomyrtone treatment were further elucidated by using transmission electron microscopy. Electron micrographs clearly showed that a newly generated daughter cell harbored the complete segregated nucleoid with midcell septum in 1% DMSO-treated cells (Fig. 6a). Thirty minutes post-treatment with 1 µg/ml rhodomyrtone led to the partial segregation of *S.* *suis* nucleoid and the septum was formed either in front of or over (guillotine effect) the incomplete-segregated nucleoid (Fig. 6b, arrow), resulting in anucleated or dead daughter cells (Fig. 6b). Cell lysis, multi-constriction of the cell membrane, asymmetrically septum formation with a consequence of asymmetric of cell division with nucleoid guillotine effects were found at 120 min post-treatment with 1 µg/ml rhodomyrtone (Fig. 6d–f). None of these abnormalities were observed in 1% DMSO control cells (Fig. 6c). The results further confirmed that rhodomyrtone treatment resulted in defective cell division by both partial nucleoid mobilization to cell pole and interfering of the Z-ring midcell-positioning.

## Discussion

Antibacterial activity of rhodomyrtone against *S.* *suis* was in the same range (0.5–2 µg/ml) in other pathogenic Gram-positive bacteria such as *S. pneumoniae*, *S. pyogenes, S. aureus* and *B. subtilis* (Limsuwan et al. 2011; Mitsuwan et al. 2017; Sianglum et al. 2011, 2018). In this study, rhodomyrtone had antibacterial potency equivalent to common and effective drug used for treatment of *S.* *suis* infection such as penicillin, ampicillin and vancomycin. Moreover, *S.* *suis* showed a lower tolerability to rhodomyrtone when compared to the relative Gram-positive bacteria such as *S.* *aureus* and *S.* *pneumoniae*. Treatment with 1 µg/ml rhodomyrtone remarkably decreased *S.* *suis* cell numbers greater than 3 log-folds within 4 h, while the same effects were observed at 6 and 8 h for *S.* *aureus* and 8 h in *S.* *pneumoniae*, respectively (Sianglum et al. 2012, 2018). This information has further endorsed rhodomyrtone as an effective narrow-spectrum antibacterial agent with low levels of MIC and MBC for treatment of Gram-positive bacterial infection. Further, application in using rhodomyrtone as a drug for treatment of *S.* *suis* zoonotic infection or elimination of *S.* *suis* from pig respiratory tract and sanitization for prevention of zoonotic infection is warrant for further investigation.

The antibacterial mechanism of rhodomyrtone at molecular level was inconclusive. Previous studies showed rhodomyrtone changed the expression of proteins playing a role in the bacterial cell cycle division. ParAB*s* system is known to play a crucial role in bacterial nucleoid segregation. Bioinformatics approaches revealed that ParAB*s* profiles were unique to the type of bacteria and tended to cluster among the phylogenetically related species and genera (Lee et al. 2003; Mohl et al. 2001; Bartosik et al. 2009). Nowadays, two closely related bacillus species, *Caulobacter crescentus* and *B. subtilis* has been used as ParABs nucleoid segregation study models (Dworkin and Losick 2002; Wang et al. 2014; Zaccaria et al. 2014) whereas the cocci including *Staphylococcus*, *Streptococcus*, and *Lactococcus* genera carried ParB (Lee et al. 2003). In this study, it was found that *S.* *suis* also carried ParB protein and *parS* DNA sequences in an absence of *parA* similar to *S.* *pneumoniae* (Ireton et al. 1994; Wang et al. 2014). ParB from *S. suis* and *S. pneumoniae* were both highly similar to *B.* *subtilis* Spo0J, the nucleoid segregation promoting protein (Wang et al. 2014). In addition, phylogenetic analysis has shown that *parS* sites in a large number of bacteria located near the *oriC* region and highly conserved for their nucleotide sequences. Three putative chromosomal *parS* sites of *S.* *suis* were found and their sequences and genetic organization were resembling to *parS* sites on *S.* *pneumoniae* chromosome (Mohl et al. 2001). These data further emphasized that the genetic components of the nucleoid segregation system were highly conserve among *Streptococci*.

Nucleoid segregation is the efficient process to ensure that each of bacterial daughter cells inherit the genetic material (Hajduk et al. 2016; Reyes-Lamothe et al. 2012; Toro and Shapiro 2010). It has been shown to be driven by the forces of bacterial general bioprocesses such as DNA replication and transcription and/or DNA-interacting proteins (Attaiech et al. 2015; Ireton et al. 1994; Lewis 2001; Strahl and Hamoen 2010). In this study, by tracking of ParB-GFP fusion protein in *S.* *suis* cells during the cell division cycle, it was demonstrated that *S.* *suis* cell division cycle spanned 60 min which was equivalent to that of *S.* *pneumoniae*. Following the complete segregation of nucleoid serving as the checkpoint, the septum formation was initiated and completed within 15 min to generate the two daughter cells of which carried a single nucleoid (chromosome), serving as a final checkpoint of cell division. Similar to those of pneumococcus (Ireton et al. 1994), *S.* *suis* nucleoid segregation covered a majority of the cell division duration. These data suggested that all coccus genera carrying ParB*s* may share the same duration and kinetic of the cell division cycle.

Dynamic of the *S.* *suis* nucleoid segregation following the rhodomyrtone treatment was investigated only for the period of 4 h due to their lack of viability after this period. Both fluorescence and EM micrographs (Figs. 4, 6) confirmed the generation of the daughter cells lacking of nucleoid or possessed the damage nucleoid from the guillotine effects. The percentages of anucleated cells we found (0.7 ± 0.2 to 3.5 ± 0.4%) were in the similar extent in pneumococci either lacking ParB or SMC functions that led to 4.7–7% guillotining nucleoid and 2–4% anucleated cells while the inhibition of the total cell transcription by inhibitors yielded 2–3% anucleated cells (Attaiech et al. 2015; Ireton et al. 1994). These data indicated that the partial interference of *S.* *suis* nucleoid mobility by rhodomyrtone seen in our study also disrupted the checkpoint of the *S. suis* cell division cycle. However, the generation of minority of the anucleated cells was not shown to be an immediate consequence to the reduction in the cell number and dead. It has been demonstrated in the growth rate determination of *parB* and/or *smc* negative mutants *S.* *pneumonia*e in liquid media bearing 2–4% anucleated cells that its cell number was not affected during the log and the stationary phases. The mutants exhibited slightly longer lag-phase compared to their isogenic wildtype. However, under the transcription inhibitor treatment, which was known to interfere the nucleoid segregation, these *S.* *pneumonia*e mutants showed a slower growth rate and hardly reached the stationary phase (Ireton et al. 1994). Thus, combinatorial accumulations of anucleated cell populations, cells with guillotine nucleoids, and inability to divide cells would compromise the cell division, thereby affected the cell number after multiple round of multiplication of which aggravated under the presence of other nucleoid segregation inhibitors. This phenomenon could also be applied to rhodomyrtone treatment in *S.* *suis* as the dramatic reduction in cell number was seen only after 4 h at the highest concentration of rhodomyrtone treatment and this duration was extended in dose-dependent manners. These might due to the combinatorial effects of rhodomyrtone on nucleoid segregation and loss of function of other proteins trapped in RIF (Saeloh et al. 2018). Therefore, the additional functional consequence of rhodomyrtone treatment on other protein which directly or indirectly involved in the nucleoid segregation remains to be elucidated.

Recently, rhodomyrtone has been demonstrated as a membrane-active compound (Saeloh et al. 2017) causing bacterial membrane potential collapse and release of intracellular proteins such as ATP and GAPDH. These indicated that the compound disturbed the intracellular bacterial protein homeostasis. Furthermore, Saeloh et al. (2018) demonstrated that rhodomyrtone caused delocalization of membrane proteins correlated to the cell size and shape defects in *B.* *subtilis* and *S.* *aureus* (Saising et al. 2018; Sianglum et al. 2018). Two important cell division proteins, DivIVA and FtsA were known to associate with FtsZ during the septum formation as a final step of cell division. In *S.* *pneumoniae,* DivIVA protein has been proposed to play a role in the control of the division site selection by ensuring FtsZ positioning at midcell compensating the absence *minCDE* operon in this organism (Fadda et al. 2007; Ni et al. 2018). Pneumococcal DivIVA interacts not only with the cell division proteins (FtsZ, FtsA, and FtsK) but also with the homolog of the nucleoid segregation protein, Spo0J protein, to cooperate both nucleoid segregation and septum formation (Fadda et al. 2007). Interestingly, the absence of *divIVA* gene in *S.* *suis* impaired the cell growth and division as evidenced by the decrease of the viable cell count, the asymmetric cell division and the aberrant of septum formation (Ni et al. 2018). Strikingly, rhodomyrtone-treated *S.* *suis* exhibited defect in both nucleoid segregation and position of septum formation leading to asymmetrical cell division resulting in anucleate mini-cells similar to that of *divIVA* gene mutation in pneumococci (Fadda et al. 2007). Although rhodomyrtone treatment resulted in trapping of a large amount of FtsZ in RIFs, it did not seem to interfere FtsZ polymerization to form Z-ring in vivo but rather the interference on its position. Therefore, it is possible that interference of rhodomyrtone treatment on nucleoid segregation was probably a consequence of the accumulation of the other membrane proteins involving in the cell division such as DivIVA or FtsA in RIFs.

## Supplementary information

**Additional file 1: Table S1.** Oligonucleotides used in this study. **Table S2.** Arbitrary fluorescence unit of *Streptococcus suis* ParB-GFP following rhodomyrtone, rifampicin or quinolone treatment, compared with 1% DMSO. **Fig. S1.***parB* DNA sequences alignment using BLASTX search. **Fig. S2.** Multiple alignment of *parS*-DNA sequences of *Streptococcus* spp.

## Data Availability

All datasets supporting the conclusion of this article are included within the article and its additional files.
