# Supplementary material for: Antibacterial mechanism of rhodomyrtone involves the disruption of nucleoid segregation checkpoint in Streptococcus suis
Source: AMB Express. 2020 Jun 8;10:110. doi: 10.1186/s13568-020-01047-x (PMC7280372; doi:10.1186/s13568-020-01047-x)
Supplement: Supplementary file 1 — Additional file 1: Table S1. Oligonucleotides used in this study. Table S2. Arbitrary fluorescence unit of Streptococcus suis ParB-GFP following rhodomyrtone, rifampicin or quinolone treatment, compared with 1% DMSO. Fig. S1.parB DNA sequences alignment using BLASTX search. Fig. S2. Multiple alignment of parS-DNA sequences of Streptococcus spp. [file 13568_2020_1047_MOESM1_ESM.docx]

**AMB EXPRESS**

**Additional file For:**

## **Antibacterial mechanism of rhodomyrtone involves the disruption of nucleoid segregation checkpoint**

## Apichaya Traithan1 · Pongsri Tongtawe1 · Jeeraphong Thanongsaksrikul1 · Supayang Voravuthikunchai2 · Potjanee Srimanote1

1 Graduate Program in Biomedical Sciences, Faculty of Allied Health Sciences, Thammasat University, Pathumthani 12120, Thailand

2 Excellent Research Laboratory on Natural Products, Department of Microbiology, Faculty of Science and Natural Product Research Center of Excellence Prince of Songkla University, Songkhla 90110, Thailand.

## Potjanee Srimanote is the corresponding author.

**Phone**: +6629869213 ext. 7218 **Fax**: +6625165379

**E-mail address**: [psrimanote01@yahoo.com.au](mailto:psrimanote01@yahoo.com.au)

**Contents:**

Table S1: Oligonucleotides used in this study

Table S2: Arbitrary fluorescence unit of *Streptococcus suis* ParB-GFP following rhodomyrtone, rifampicin or quinolone treatment, compared with 1% DMSO

Fig. S1: *parB* DNA sequences alignment using BLASTX searc

Fig. S2: Multiple alignment of *parS*-DNA sequences of *Streptococcus spp.*

**Table S1** Oligonucleotides used in this study

| **Name** | **Sequence (5’** 🡪 **3’)*** |
| --- | --- |
| Us*parB*-F+*Sph*I | GGCATGCTAGCTGTTGCTGGAGAGG |
| Ds*parB*-R+*Bam*HI | CTGGGGATCCCCCATTTGAAACGTGTTAC |
| *gfp*-F | CGAAAGAATTATCAACATGAGTAAAGGAGAAGAACTTTTC |
| *gfp*-R | GGAAAAGAGTGTTTACAGTTGGAATTCATTATTTGTAG |
| *parB*OE | CTTCTCCTTTACTCATGTTGATAATTCTTTCG |
| *gfp*OE | TAATGAATTCCAACTGTAAACACTCTTTTCC |
| pSET4s-F (vector primer) | AGATCTCGGTGATGACGGTGAAAACC |
| pSET4s-R (vector primer) | ACTAGTTATCTACACGACGGGG |

## * Restriction sites were underlined.

**Table S2** Arbitrary fluorescence unit of *Streptococcus suis* ParB-GFP following rhodomyrtone, rifampicin or quinolone treatment, compared with 1% DMSO

| **Test** | **Determination percentage of arbitrary fluorescence unit, compared with the untreated at different time-points (h)a** | | | | | |
| --- | --- | --- | --- | --- | --- | --- |
|  | **0** | **1** | **2** | **3** | **4** | **5** |
| **Rhodomyrtone** | | | | | | |
| 2 × MIC | 76 ± 8** | 66 ± 12** | 64 ± 9** | 37 ± 2** | -5 ± 2** | -10 ± 4** |
| 1 × MIC | 86 ± 6 | 74 ± 7** | 72 ± 8** | 48 ± 7** | 11 ± 3** | 0 ± 6** |
| 0.5 × MIC | 96 ± 7 | 84 ± 10 | 71 ± 8** | 71 ± 4** | 43 ± 5** | 30 ± 4** |
| 0.25 × MIC | 99 ± 7 | 93 ± 10 | 87 ± 8 | 84 ± 2** | 62 ± 2** | 35 ± 3** |
| 0.125 × MIC | 99 ± 7 | 93 ± 10 | 89 ± 7 | 83 ± 5** | 68 ± 3** | 56 ± 6** |
| **Rifampicin** | | | | | | |
| 2 × MIC | 85 ± 6** | 79 ± 10** | 74 ± 8** | 54 ± 2** | 34 ± 4** | 17 ± 5** |
| 1 × MIC | 97 ± 5 | 95 ± 7 | 83 ± 8* | 71 ± 2** | 44 ± 5** | 29 ± 4** |
| 0.5 × MIC | 95 ± 6 | 90 ± 9 | 88 ± 7 | 77 ± 3** | 61 ± 4** | 31 ± 6** |
| 0.25 × MIC | 95 ± 7 | 94 ± 8 | 93 ± 7 | 83 ± 4** | 69 ± 5** | 48 ± 4** |
| 0.125 × MIC | 96 ± 7 | 95 ± 9 | 91 ± 8 | 90 ± 2 | 74 ± 5** | 56 ± 5** |
| **Quinolone** | | | | | | |
| 2 × MIC | 65 ± 7** | 60 ± 11** | 43 ± 9** | 25 ± 3** | -7 ± 4** | -24 ± 3** |
| 1 × MIC | 77 ± 8** | 68 ± 9** | 59 ± 8** | 46 ± 3** | -1 ± 4** | -12 ± 4** |
| 0.5 × MIC | 84 ± 7** | 76 ± 8** | 66 ± 8* | 59 ± 1** | 40 ± 3** | 22 ± 6** |
| 0.25 × MIC | 86 ± 8 | 80 ± 9** | 73 ± 10* | 64 ± 3** | 49 ± 4** | 32 ± 5** |
| 0.125 × MIC | 98 ± 6 | 94 ± 8 | 88 ± 8 | 80 ± 4** | 61 ± 2** | 45 ± 2** |

a Fluorescence was reported in arbitrary units and was determined as described in Materials and Methods.

* The significance drug treatment and concentrations that affect to the GFP activity at different time were analyzed using the Student’s t-test. p-values < 0.05 (*) and p-values < 0.001 (**) were considered statistically significant.

**a**


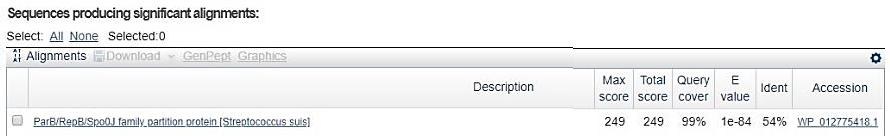


**b**


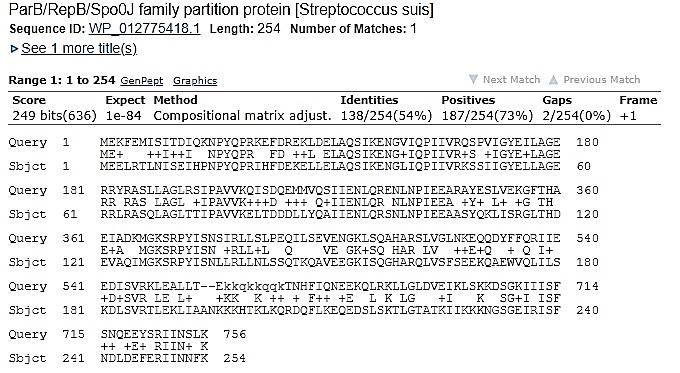


**Fig. S1** *parB* DNA sequences alignment using BLASTX search (**a**) (Available from [http://blast.ncbi.nlm.nih.gov/Blast.cgi;](http://blast.ncbi.nlm.nih.gov/Blast.cgi%3B) Accessed on August 26, 2015). Multiple alignments of deduced amino acid sequences of ParB (**b**). The consensus sequence from P1/7 *S. suis* was analyzed by comparing with ParB amino acid residues of D39 *S. pneumoniae* (GenBank accession no. [WP_000410378.1](https://www.ncbi.nlm.nih.gov/protein/446332523))

**a**


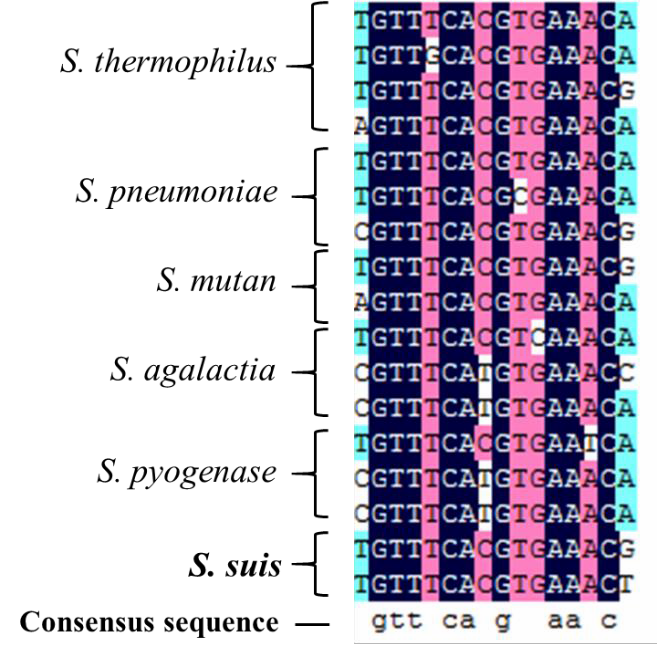


# b


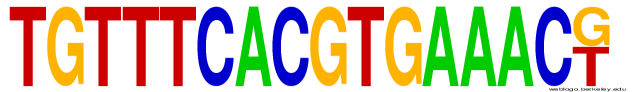


**Fig. S2** Multiple alignment of *parS*-DNA sequences of *Streptococcus spp.* included *S. suis* (**a**) and WebLogo showing the consensus of *parS* sites in *S. suis* wildtype P1/7 (**b**)
